# Potential Association Between Acute Colonic Pseudo-Obstruction (Ogilvie Syndrome) and Oral Nimodipine: Report of Two Cases

**DOI:** 10.7759/cureus.28039

**Published:** 2022-08-15

**Authors:** Orlando De Jesus, Jose Sánchez Jiménez, Juan C Vicenty

**Affiliations:** 1 Neurosurgery, University of Puerto Rico, Medical Sciences Campus, San Juan, PRI

**Keywords:** colon distention, aneurysmal subarachnoid hemorrhage, acute colonic pseudo-obstruction, ogilvie syndrome, nimodipine

## Abstract

Nimodipine is a calcium channel blocker used for the management of patients with aneurysmal subarachnoid hemorrhage. Oral nimodipine has been rarely implicated in the development of acute colonic pseudo-obstruction (Ogilvie syndrome) in patients treated for aneurysmal subarachnoid hemorrhage. Nimodipine inhibits the transmembrane influx of calcium ions which are essential for the excitation-contraction coupling process of smooth muscle cells. We thought this mechanism of action could predispose patients to develop Ogilvie syndrome. This report aimed to examine the existing literature concerning the potential association between Ogilvie syndrome and nimodipine in patients with aneurysmal subarachnoid hemorrhage. All published cases of aneurysmal subarachnoid hemorrhage associated with Ogilvie syndrome were reviewed. We presented two female patients with aneurysmal subarachnoid hemorrhage produced after a ruptured anterior communicating artery aneurysm who received oral nimodipine and developed Ogilvie syndrome. The patients developed Ogilvie syndrome four to six days after receiving oral nimodipine. These two cases may further support the potential association of Ogilvie syndrome with the use of oral nimodipine during the treatment of patients with aneurysmal subarachnoid hemorrhage.

## Introduction

Ogilvie syndrome, also known as acute colonic pseudo-obstruction, is the distension of the colon caused by reduced motility in the absence of mechanical obstruction [1,2]. The most common risk factors associated with its development include severe infection, severe disease process, myocardial infarction, congestive heart failure, major surgical procedures, metabolic disorders, electrolyte disorders, renal insufficiency, systemic lupus erythematosus, gastrointestinal carcinoma, severe trauma, spinal cord injury, cerebral stroke, Guillain-Barré syndrome, Parkinson’s disease, some medications, and alcohol abuse. Nimodipine, a second-generation dihydropyridine type calcium channel antagonist that blocks the influx of extracellular calcium through L-type voltage-gated calcium channels, is indicated in the management of patients with aneurysmal subarachnoid hemorrhage (SAH) [3-5]. Oral nimodipine is considered a safe drug with only minor side effects, including a minimal transient drop in systemic blood pressure and reversible liver enzyme increase [3].

Oral nimodipine has been rarely associated with the development of acute colonic pseudo-obstruction, with only one reported case [6]. Using a case series of two patients, we aimed to describe the potential association between acute colonic pseudo-obstruction and nimodipine. This report presented two patients who had aneurysmal SAH and developed acute colonic pseudo-obstruction while receiving oral nimodipine. The available literature was reviewed to determine the potential association between nimodipine used for aneurysmal SAH and Ogilvie syndrome. This article was previously posted to the Research Square preprint server on August 10, 2021.

## Case presentation

Case 1

A 61-year-old female with a history of hypertension presented to the local health center after a sudden severe headache. She was alert and oriented with mild neck rigidity. A head computed tomographic (CT) scan showed a thick diffuse basal cisternal SAH with a small amount of intraventricular hemorrhage (modified Fisher grade four). In view of the findings, she was intubated before being transferred to our institution. On arrival at our institution, she had a blood pressure of 174/97 mm Hg, intubated but alert and oriented, and following commands. The patient was immediately started on oral nimodipine 60 mg orally every four hours. A cerebral digital subtraction angiogram (DSA) was performed the following day, which showed a small anterior communicating artery aneurysm measuring 2.7 x 2.8 x 4.2 mm with a neck of 2.9 mm filling primarily through the left anterior cerebral artery. During the procedure, she was embolized using detachable helical coils with the complete occlusion of the aneurysm, and then transferred to the intensive care unit and extubated. Post extubation, the patient had no neurological deficits.

Two days after her admission, she developed a distended abdomen but was afebrile. She tolerated the diet without nausea, vomiting, or diarrhea and passed gas and solid stools. A plain abdominal X-ray showed a distended large bowel with no evidence of mechanical obstruction. As the distended abdomen persisted for the next two days, an abdominal CT scan was performed which showed a 10 cm dilatation of the ascending and transverse colon without an obstructive lesion or evidence of a sharp transition point. She presented the classic radiological presentation of acute colonic pseudo-obstruction, excluding other similar clinical differentials like ileus, mechanical obstruction, toxic megacolon, volvulus, and ischemic colitis. She had been on oral nimodipine for four days.

**Figure 1:**
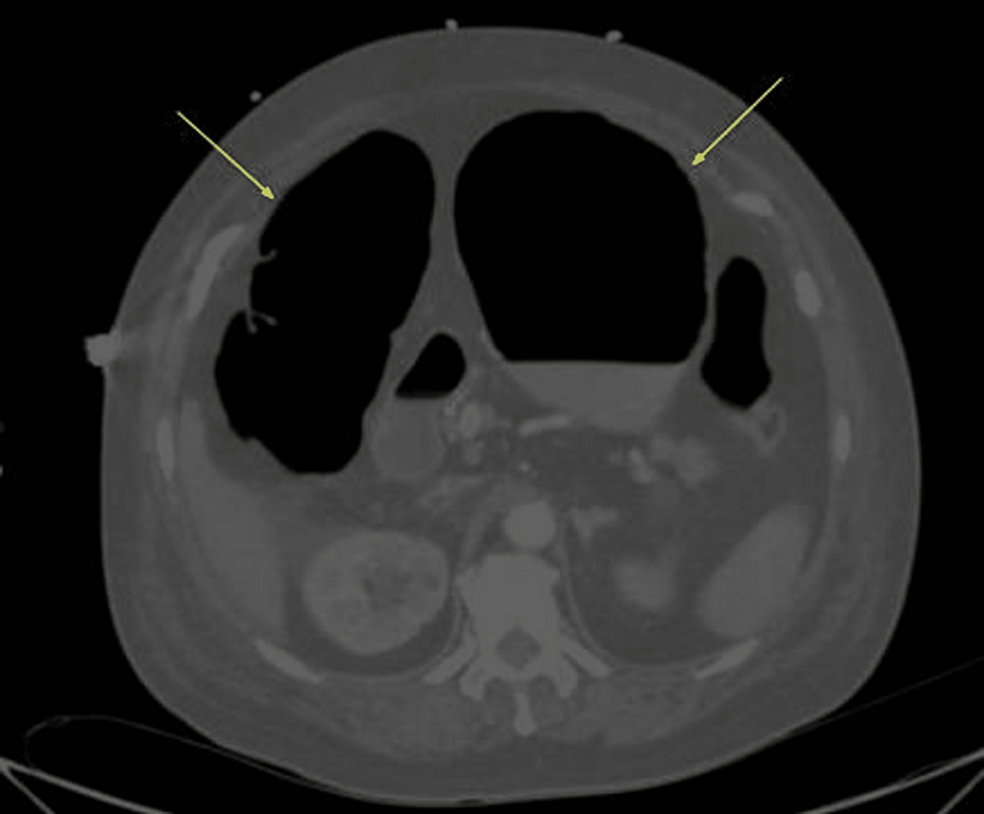
Abdominal CT scan axial image shows a 10 cm dilatation involving the ascending and transverse colon (yellow arrows)

The diet was suspended, including oral nimodipine, and a nasogastric tube was placed with intermittent suction for five days; however, the abdominal distension did not improve. Neostigmine 2 mg was given intravenously over three to five minutes and repeated the following day. A brain CT angiography showed moderate vasospasm with a 50% reduction in the diameter of the anterior cerebral vessels. Although she was asymptomatic from the vasospasm, she had been five days without nimodipine. Given the possibility of delayed ischemic complications, nimodipine was restarted. A repeat abdominal CT scan was performed, which showed an interval reduction of the dilatation measuring 7 cm at the ascending and transverse colon with a normal descending colon, sigmoid, and rectum. The diet was restarted two days later, but she developed significant abdominal distension, so the diet was paused. A repeat cerebral DSA revealed substantial improvement in the vasospasm. Thus, it was decided not to continue the nimodipine. As the abdominal distension had not improved, a colonoscopy for decompression was attempted, but decompression was unsuccessful. A rectal tube was then placed with an output of 850 ml in 24 hours. The abdominal distension rapidly improved, and the tube was removed the following day. The diet was then restarted. Four days later, a plain abdominal X-ray showed resolution of the colon distension. Five days after the rectal tube was removed and 28 days after diagnosing the colonic pseudo-obstruction, she was discharged home.

Case 2

A 46-year-old female with a history of bipolar disease and heart arrhythmias with a pacemaker had a sudden severe headache and arrived at our emergency department for evaluation. Neurological examination showed an alert and oriented patient without neurological deficit. The head CT scan showed a thin anterior interhemispheric SAH (modified Fisher grade one), and the CT angiography showed an anterior communicating artery aneurysm. The patient was immediately started on oral nimodipine 60 mg orally every four hours. A cerebral DSA was performed the following day, which confirmed the presence of an anterior communicating artery aneurysm measuring 3.9 x 4.5 x 4.4 mm with a neck of 4.5 mm filling primarily through the left anterior cerebral artery. Embolization of the aneurysm was not recommended because of the wide neck and a neck-to-dome ratio of one. She was taken to the operating room for a craniotomy and clipping of the aneurysm. Just after the opening of the dura mater, the patient had an episode of rebleeding. A ventriculostomy was placed, which relaxed the brain, and the aneurysm was successfully clipped. Postoperative, she continued to be alert and oriented without neurological deficit.

Five days after admission, she developed a distended abdomen. She was afebrile. The diet was tolerated without nausea, vomiting, or diarrhea. She passed gas and solid stools. A plain abdominal X-ray showed distension of the large bowel with no evidence of mechanical obstruction. An abdominal CT scan was performed the next day, which showed a 9.5 cm dilatation of the transverse colon and sigmoid without an obstructive lesion. The clinical presentation and radiological findings were compatible with acute colonic pseudo-obstruction. She presented the classic radiological presentation of acute colonic pseudo-obstruction, excluding other similar clinical differentials like ileus, mechanical obstruction, toxic megacolon, and ischemic colitis. She had been using oral nimodipine for six days.

**Figure 2:**
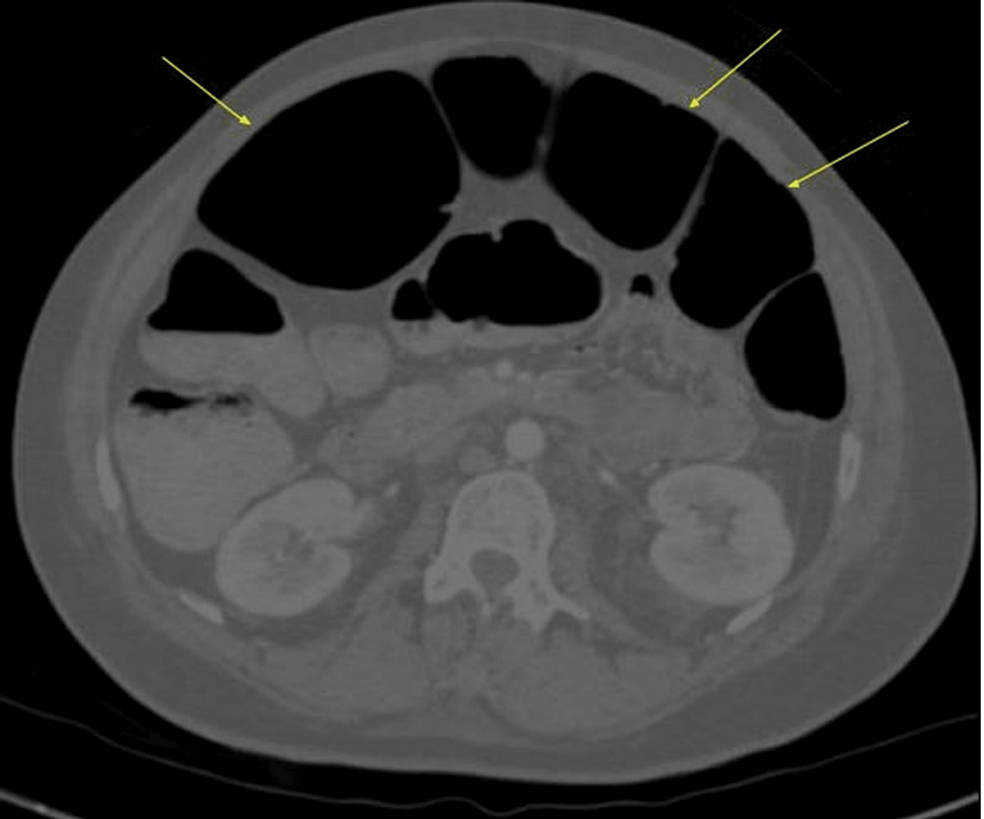
Abdominal CT scan axial image shows a maximum dilatation of 9.5 cm at the transverse colon and sigmoid (yellow arrows)

The diet was immediately stopped, including oral nimodipine, and a nasogastric tube was placed with intermittent suction for two days. However, the abdominal distension did not improve, and a rectal tube was placed two days later with a large output in 24 hours. The abdominal distension decreased significantly. The tube was removed the following day, and she was started again on a diet. Nimodipine was restarted to complete the 21 days of treatment. Two days later, a plain abdominal X-ray showed a reduction in the distension of the colon. Seven days after the rectal tube was removed and twelve days after diagnosing the colonic pseudo-obstruction, she was discharged home. Six months later, a ventriculoperitoneal shunt was placed after the development of hydrocephalus.

## Discussion

During the early 1980s, nimodipine was considered the drug of choice for preventing and treating cerebral vasospasm following SAH due to its preferential cerebrovascular action [4,5]. It is now recognized that oral nimodipine improves the clinical outcome in patients with delayed cerebral ischemia after SAH secondary to intracranial arterial spasm [7,8]. Nimodipine blocks the influx of extracellular calcium. The transmembrane influx of calcium ions is essential for the excitation-contraction coupling process of smooth muscle cells. Specific antagonists of this calcium influx can potentially inhibit smooth muscle contraction. Experimental evidence of inhibitory effects of calcium antagonists on intestinal smooth muscle contraction had been previously published [9,10]. These experimental data provided evidence that calcium antagonists exert inhibitory effects on the gastrointestinal tract due to their intrinsic mechanism of action.

In 1948, Ogilvie reported two patients with signs of colonic obstruction without any organic disease of the colon [1]. He attributed its development to interrupting the sympathetic supply to the large intestine. After surgical exploration, both patients were found to have disseminated abdominal metastatic disease with infiltration of the ganglia but no mechanical obstruction of the colon. Ogilvie syndrome is now recognized as a colonic pseudo-obstruction caused by reduced motility without mechanical obstruction. Ogilvie syndrome is associated with a 25-30% mortality in severe cases, increasing up to 50% if the patient develops ischemia and perforation [2]. Prompt recognition and management of the syndrome are essential. The specific pathophysiology of acute colonic pseudo-obstruction is still unclear. It may include many etiologies, risk factors, pathological conditions, and multiple associations leading to altered autonomic regulation of colonic motility [2,11]. Excessive parasympathetic suppression, sympathetic stimulation, or both are thought to produce an imbalance in the colonic autonomic innervation. These actions temporarily suppress intestinal motility, inducing the acute dilatation of the colon. 

Nimodipine used for aneurysmal SAH has been rarely associated with the development of Ogilvie syndrome. In 1987, Torrealba et al. were the first to describe a patient with acute colonic pseudo-obstruction who received intravenous nimodipine for a SAH whose condition improved after a rectal tube was inserted and the nimodipine was discontinued [12]. However, no cerebral aneurysm was identified in the angiographic studies. Hund et al. reported a similar complication after using intravenous nimodipine in a patient who had a SAH secondary to a ruptured aneurysm of the anterior communicating artery [13]. They also mentioned four prior patients with aneurysmal SAH who developed abdominal distension after intravenous nimodipine [13].

Fahy described a patient receiving oral nimodipine therapy for a SAH secondary to an aneurysm of the posterior communicating artery who developed acute colonic pseudo-obstruction [6]. This report was the first to associate the oral use of nimodipine with the development of Ogilvie syndrome. The patient continued treatment with nimodipine for the recommended time, despite the colonic distension, as it was thought the risk of removing the medication could be more detrimental than its maintenance. The pseudo-obstruction resolved after endoscopic colon decompression and rectal tube placement. For one of our patients, we performed similar management to improve the pseudo-obstruction. Our other patient did not require a colonoscopy as she improved with a rectal tube. Our two cases provide additional evidence about the potential causal association between the use of nimodipine and Ogilvie syndrome in patients treated for aneurysmal SAH. A limitation of this report is that only two patients in our institution developed Ogilvie syndrome among those treated with nimodipine for aneurysmal subarachnoid hemorrhage. However, this association could be an uncommon phenomenon considering the large number of patients who receive oral nimodipine following aneurysmal subarachnoid hemorrhage that do not develop Ogilvie syndrome.

Patients with Ogilvie syndrome are initially managed with supportive therapy to decompress the gastrointestinal tract, including nasogastric and rectal tubes. Conservative treatments include discontinuation of the oral intake, placement of a nasogastric tube for proximal gut decompression, correction of fluid and electrolyte abnormalities, treatment of any underlying concomitant illnesses, and the cessation of medications such as narcotics and anticholinergics that adversely affect colonic motility [14]. Those who do not respond within two days may require intravenous neostigmine. Intravenous neostigmine, an acetylcholinesterase inhibitor, is the best-documented pharmacological treatment for acute colonic pseudo-obstruction, with initial response rates of 60-90% [11,14-16]. Although neostigmine has a short elimination half-life, most patients in the study by Ponec et al. achieved a sustained response after the initial dose [15]. We used neostigmine in one of our patients; however, it only partially improved the abdominal distension, reappearing when the patient was restarted on a diet. Haj et al. showed similar outcomes using conservative or interventional management to treat Ogilvie syndrome [17]. Patients who do not respond to conservative treatment should receive endoscopic decompression [14]. Colonoscopic decompression is successful in approximately 80% of patients. Exploratory laparotomy is limited to patients with complications [14].

## Conclusions

The use of nimodipine for aneurysmal SAH may predispose patients to develop Ogilvie syndrome. The continuation of nimodipine after developing Ogilvie syndrome has not been established, and each physician should decide based on the risk of withdrawing the medication. Additional investigation into the influence of oral nimodipine on the gastrointestinal tract of patients with aneurysmal SAH is recommended.
